# Where You Lead, I Will Follow: Exploring Sibling Similarity in Brain and Behavior During Risky Decision Making

**DOI:** 10.1111/jora.12581

**Published:** 2020-09-18

**Authors:** Christy R. Rogers, Tae‐Ho Lee, Cassidy M. Fry, Eva H. Telzer

**Affiliations:** ^1^ Texas Tech University; ^2^ Virginia Polytechnic Institute and State University; ^3^ The Pennsylvania State University; ^4^ University of North Carolina at Chapel Hill

## Abstract

This exploratory study examined whether social learning increases similarity in adolescent siblings’ behavior and neural patterns during risky decision making. Participants included 86 adolescents (43 sibling dyads; younger siblings: *M*_age_ = 12.2 years; 22 females; older siblings: *M*_age_ = 14.6 years; 20 females) who completed questionnaires, and a decision‐making task during an fMRI scan. Younger siblings became more similar to their older siblings’ risky decision making after observing their older sibling take risks). Younger siblings who reported greater modeling of their older sibling, and less differentiation from them, showed increased neural similarity to their older siblings in the ventromedial prefrontal cortex, and the right anterior insula and ventral striatum, respectively. These findings highlight siblings as salient social agents in how adolescents process risky decision making.

Risky decision making and sensation seeking rapidly increase during adolescence (Steinberg et al., 2018), leading to greater engagement in behaviors such as substance use, risky sexual activity, and dangerous driving (Chambers, Taylor, & Potenza, 2003; Defoe, Dubas, Figner, & van Aken, 2015; Steinberg et al., 2018; Young et al., 2002). As adolescents navigate novel goals, such as thrill‐seeking or impressing a peer, their behavior is highly contingent on social contexts (Knoll, Magis‐Weinberg, Speekenbrink, & Blakemore, 2015; Kuhn, 2006). One way that adolescents learn how to act and behave occurs through the observation of those around them (e.g., Akers & Lee, 1996), a process called social learning (Bandura, 1977). Exposure to social norms (e.g., drinking behavior) sets the stage for what behaviors are valued, appropriate, and desirable, and the more adolescents are exposed to and learn the value attached to these behaviors, the more they internalize those norms and model the behavior in future instances (Akers et al., 1979). Perspectives from social neuroscience propose that adolescents are particularly sensitive to social contexts during decision making (Knoll, Magis‐Weinberg, Speekenbrink, & Blakemore, 2015; Schriber & Guyer, 2016; Somerville et al., 2010; Steinberg, 2008) due to reorganization in the brain during the transition from childhood to adolescence (Blakemore & Mills, 2014; Nelson, Jarcho, & Guyer, 2016; Shulman et al., 2016). Research has linked the importance of parental (Guassi Moreira & Telzer, 2018; Qu, Fuligni, Galvan, Lieberman, & Telzer, 2016; Qu, Fuligni, Galvan, & Telzer, 2015; Telzer, Ichien, & Qu, 2015) and peer (Chein et al., 2011; Telzer, Fuligni, Lieberman, Miernicki, & Galván, 2015; Telzer, Miernicki, & Rudolph, 2018; Vorobyev, Kwon, Moe, Parkkola, & Hämäläinen, 2015) influence on adolescent neural activity during risky decision making (for a review, Telzer et al., 2017), yet the behavioral and neural effects of social learning have yet to be investigated.

Although prior research has largely focused on the role of peers, sibling relationships are a salient influence on whether adolescents decide to engage in risk taking (Rende, Slomkowski, Lloyd‐Richardson, & Niaura, 2005; Slomkowski, Rende, Novak, Lloyd‐Richardson, & Niaura, 2005; Whiteman, Jensen, & Maggs, 2013). While sibling relations can be a protective factor in promoting psychosocial adjustment during adolescence (Branje, van Lieshout, van Aken, & Haselager, 2004; Gass, Jenkins, & Dunn, 2007; Hollifield & Conger, 2015; Rogers, Guyer, Nishina, & Conger, 2018), they also have the ability to increase adolescent engagement in risk taking (Craine, Tanaka, Nishina, & Conger, 2009; Slomkowski, Rende, Conger, Simons, & Conger, 2001; Whiteman, Zeiders, Killoren, Rodriguez, & Updegraff, 2014). In fact, older sibling risk taking predicts younger siblings’ risk taking above and beyond the influence of parents and peers (Defoe et al., 2013; Stormshak, Comeau, & Shepard, 2004), even among nonbiological siblings (McGue & Sharma, 1995; Samek, Rueter, Keyes, Mcgue, & Iacono, 2015). Sibling influence occurs above and beyond the effects of early child aggression, maternal mental health, marital status, family violence, parental criminality, and family income, suggesting that concordance in siblings’ risk taking cannot be fully explained by shared experiences or genetics, but must be attributed to the sibling relationship itself (Fagan & Najman, 2003; Slomkowski et al., 2005). Importantly, sibling social connectedness influences adolescent risk taking, and such effects are not driven by genetic similarity (Slomkowski et al., 2005), underscoring siblings as an ideal relationship to test the effects of social learning in a risk‐taking context (e.g., Whiteman, Zeiders, Killoren, Rodriguez, & Updegraff, 2014). Sibling relationships serve as a training ground for aggressive and deviant behavior (e.g, Garcia, Shaw, Winslow, & Yaggi, 2000; Patterson, Dishion, & Bank, 1984) in which adolescents observe, learn, and practice the negative behaviors carried out by their older siblings (for a review, McHale, Updegraff, & Whiteman, 2012; Whiteman, McHale, & Crouter, 2007a), consistent with social learning theory (Bandura, 1977). Given that older siblings tend to have more privileges (e.g., freedom with friends, later curfew), greater access to resources (e.g., money, alcohol), and more life experiences (e.g., dating, substance use), they can serve as salient antisocial models via social learning (Feinberg, Solmeyer, & McHale, 2012), and thus, reinforce risky attitudes and empower the risky behaviors of their younger siblings (Slomkowski et al., 2001; Snyder, Bank, & Burraston, 2005).

While many theories have been proposed to describe neurobiological sensitivity to social context (Schriber & Guyer, 2016), no empirical study to date has implemented fMRI to identify changes in the brain as a result of social learning from salient social models during risk taking, despite the evidence suggesting the pivotal role social learning plays in adolescent decision making (see Telzer, van Hoorn, Rogers, & Do, 2018). Nonetheless, emerging research has begun to highlight how peers (Chein et al., 2011; Telzer, Ichien, & Qu, 2015; Telzer, Miernicki, et al., 2018; van Hoorn, McCormick, Rogers, Ivory, & Telzer, 2018; Vorobyev et al., 2015), parents (Guassi Moreira & Telzer, 2018; Qu et al., 2016, 2015; Telzer, Ichien, et al., 2015; Telzer, Fuligni, et al., 2015; van Hoorn et al., 2018), and even siblings (Rogers, McCormick, van Hoorn, Ivory, & Telzer, 2018) influence adolescent neurodevelopment and risk taking (for a meta‐analysis, see van Hoorn, Shablack, Lindquist, & Telzer, 2019). These studies highlight a set of neural candidates to examine as promising indices of adolescent susceptibility to social influence. The ventral striatum (VS; for a review, Fareri, Martin, & Delgado, 2008) and anterior insula (AI; for a review, Smith, Steinberg, & Chein, 2014) are implicated in adolescent risky decision making (Kahn, Peake, Dishion, Stormshak, & Pfeifer, 2015; McCormick & Telzer, 2017; Peake, Dishion, Stormshak, Moore, & Pfeifer, 2013; Telzer, Ichien, et al., 2015; Telzer, Fuligni, et al., 2015;), and activation in these regions is modulated by changes in the social context (Chein et al., 2011; Qu et al., 2015; Rogers, Guyer, Nishina, & Conger, 2018; Telzer, Ichien, et al., 2015; Telzer, Fuligni, et al., 2015; Telzer, Miernicki, et al., 2018). In addition, the ventromedial prefrontal cortex (vmPFC) is implicated in valuation (Hare, Camerer, & Rangel, 2009), specifically as adolescents learn during risky decision making (for a review, Blakemore & Robbins, 2012). Together, the VS, AI, and vmPFC represent regions associated with reward value and the integration of reward‐related decision making in a risk‐taking context.

Most prior research examining the neural processes involved in susceptibility to social influence has tested how the presence of others (e.g., peers, parents, unknown adults) changes adolescents’ neural processing and subsequent risky behavior (Chein et al., 2011; Guassi Moreira & Telzer, 2018; Telzer, Ichien, et al., 2015; Telzer, Fuligni, et al., 2015). While this research has significantly increased our understanding of how adolescent risk taking is altered in different social contexts, it does not examine the cognitive‐behavioral effects of social learning. Social learning is a multifaceted process involving observation, identifying the attitudes and salience of the model, assessing the cost‐benefit ratio of performing the behavior, and making subsequent decisions (Akers, 2011), and has been identified as a key mechanism underlying adolescent risk taking (e.g., Pomery et al., 2005; Rende et al., 2005; Stormshak et al., 2004; Whiteman et al., 2014). Given that no prior behavioral or neuroimaging research has implemented experimental designs to examine social learning processes in vivo, we conducted an exploratory study in which we implemented a novel manipulation to examine the effect of complex social learning processes involved in social influence susceptibility. In particular, younger and older siblings each completed a task that measures risky decision making in an uncertain context during fMRI. The younger siblings then observed their older siblings’ risk behavior on the same task and then completed the task again. This manipulation has two novel components. First, we can examine changes in the younger siblings’ own risky behavior in the task and associated brain circuitry as a function of social learning (i.e., observation of their older sibling engaging in risk taking). Second, by scanning the siblings as they complete the same task, we can implement innovative techniques to test neural similarity between siblings. A novel approach to investigating how dyads (e.g., friend pairs: Parkinson, Kleinbaum, & Wheatley, 2017, 2018; parent–child pairs: Lee, Miernicki, & Telzer, 2017; Lee, Qu, & Telzer, 2018) influence decision making and neural processing includes examining dyadic neural similarity, which identifies similarity within specific regions of interest (ROI) in the brain. For our purposes, neural similarity between siblings can capture the extent to which social learning can induce greater similarity in the recruitment of the same brain regions between siblings as they make risky decisions.

In this exploratory study, we hypothesized that younger siblings would become more similar to their older sibling following social learning. At the behavioral level, this would be evident by younger sibling behavior changing in the direction of older sibling behavior following social learning. At the neural level, we hypothesized that sibling neural similarity would increase in the VS, vmPFC, and AI following social learning because adolescents may process the valuation and salience of risky decisions more similarly to their older sibling. Specifically, we expected a significant difference in activation patterns between siblings from baseline to postsocial learning, such that sibling neural similarity would be low at baseline and moderate after social learning.

Although we tested for mean level changes, we did not expect all siblings to show strong social learning effects, but that this would be modulated by characteristics of their relationship. Because the salience of a given model is integral to social learning (Akers, 2011), we investigated the social learning processes of sibling modeling and differentiation. Sibling modeling is the process by which younger siblings observe and willingly replicate the behavior of their older sibling because they hold the model in high esteem and perceive the behavior as valuable (Whiteman et al., 2014). Differentiation is the process through which adolescents actively differentiate from their older sibling to establish a unique identity and reduce social comparison (Whiteman, Becerra, & Killoren, 2009). The agency to process the social behavior and choose to be different from another individual is an important part of social learning (Bandura, 2001), as adolescents incorporate new social information and assess its relevance to previous experiences (Akers, 2011). Thus, we hypothesized that greater modeling and less differentiation would be associated with greater similarity between younger and older siblings’ risky behavior, as suggested by previous research (Whiteman et al., 2013), and greater similarity between their neural patterns in the VS, AI, and vmPFC following social learning. Of note, previous research has found stronger effects of sibling influence on adolescent risk taking for same‐sex dyads (Slomkowski et al., 2001) and characteristics of the shared environment (i.e., parent involvement; Samek, Rueter, Keyes, Mcgue, & Iacono, 2015), but due to the exploratory nature and small sample size of the current study, we focused on the processes of modeling and differentiation.

## Methods

### Participants

Participants included 86 adolescents, comprised of 43 sibling dyads with a younger sibling (*M*_age_ = 12.2 years, range = 11–14; 22 females) and their older sibling (*M*_age_ = 14.6 years, range = 13–17; 20 females). Older siblings were within 4 years of age from the younger sibling, as social learning most often occurs within siblings who are closer in age (McGue & Sharma, 1995), and this age‐range is the common practice in family studies (e.g., Craine et al., 2009; Whiteman, Jensen, Mustillo, & Maggs, 2016). Sibling dyad age difference ranged from 1.19 to 4.28 years, and the sex constellation was as follows: 9 sisters, 10 brothers, 13 older brother younger sister pairs, and 11 older sister younger brother pairs. Participants were recruited through community flyers and social media postings (i.e., Facebook, Craigslist). Adolescent ethnicity, family socioeconomic status, and parental marital status are displayed in Table 1. Inclusion criteria included that the older sibling lived in the home for the duration of the younger sibling’s life (sibling dyad relatedness: *n = *39 full biological; *n* = 2 half biological; *n* = 2 adopted) and that both adolescent participants were free from MRI contraindications, learning disabilities, diagnosis of ADHD, and neurological‐altering medications. Two additional adolescents were excluded from analyses because they did not complete the scan due to claustrophobia, and thus, two sibling dyads were excluded (full sample included 45 sibling dyads). All participants provided written assent with parental consent in accordance with the Institutional Review Board.

**Table 1:** Demographics of Adolescent Ethnicity, Family Total Income, Parental Education, and Parental Marital Status

Variables	*n*
Adolescent ethnicity (*N* = 86)	
Latino/Hispanic	7
African American/Black	7
Asian American/Pacific Islander	2
Caucasian/White	61
Multiethnic	9
Family total income (*N* = 43)	
<$45,000	2
$45,000–$74,999	11
$75,000–$99,999	12
$100,000–$150,000	11
>$150,000	6
Parental education (*N* = 43)	
Some high school	1
High school diploma	1
Some college	5
Associate’s degree	6
Bachelor’s degree	12
Some graduate school	3
Master’s degree (e.g., M.A., MS.W.)	12
Professional degree (e.g., M.D., Ph.D.)	3
Parental marital status (*N* = 43)	
Single	3
Married to first spouse	34
Divorced and remarried	6

### Procedure

Younger and older sibling participants attended the scan session on the same day with a participating parent. After the consent and assent process, an experimenter escorted the parent to a separate room to complete questionnaires. Next, a second experimenter accompanied the older sibling to a separate room to complete task training on a laptop and then to the scanner for their scan session. During this time, younger siblings completed a series of questionnaires and task training on a laptop with a third experimenter. Following the older sibling’s scan, the younger sibling completed their scan, while the older sibling completed a series of questionnaires in a separate room. To ensure that the sibling dyad did not come into contact with one another while moving to and from the scanner, separate hallways and rooms within the scanner suite were utilized throughout the duration of the session. The total session length lasted an average of 3.5 hours, though the duration varied between families.

### Sibling Influence Scale

Younger siblings completed the sibling influence scale (Whiteman, Bernard, & McHale, 2010), which assesses how influential the participating older sibling is on the adolescent’s behavior. The scale comprises of 18 items for which adolescents reported on a scale from 1 = *never* to 5 = *very often* on behaviors that occur when they spend time with their older sibling. The sibling influence scale includes two subscales: eight items for modeling, which encompasses positive modeling behaviors demonstrated by the older sibling to the adolescent, as well as adolescent learning via older sibling modeling (e.g., “My sibling sets an example for how I should behave); and 10 items for differentiation, which reflects adolescent behavior and attitudes to avoid similarity with their older sibling (e.g., “I try to make different choices than my sibling”). Items for each subscale were summed and the items for differentiation were reverse‐scored such that higher scores indicated less differentiation (α = .82), and high levels of modeling (α = .73). Because modeling and differentiation were not significantly correlated (*r* = .24, *p* = .118), these processes were analyzed separately, given that they are distinct processes of sibling influence (Whiteman et al., 2007a).

### Risky Decision‐Making Task

**Figure 1:**
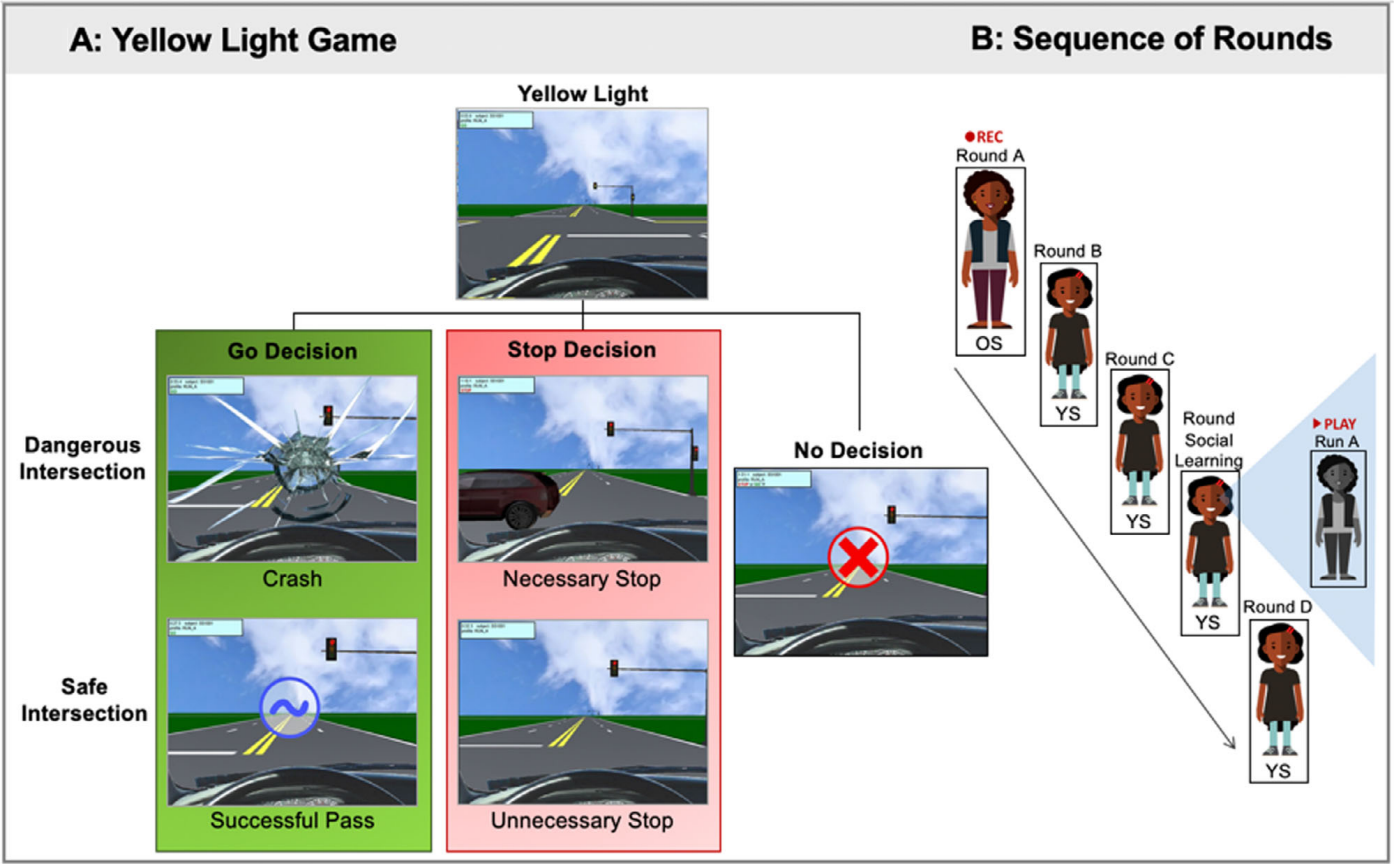
(a) Task paradigm of the Yellow Light Game (YLG). (b) Sequence in which adolescents completed the YLG. OS = older sibling; YS = younger sibling.

Participants completed the Yellow Light Game (YLG; Op de Macks et al., 2018), a virtual driving simulation adapted from the Stoplight task (Chein et al., 2011; Steinberg et al., 2008), to examine both behavior and the neural processing of risky decision making. However, the task was only referred to as the “driving game” to participants, who were not explicitly informed about the risk‐taking or social learning goals of the study. Participants were instructed to complete the driving course as fast as possible, choosing to either go or stop at each yellow light intersection (Figure 1a). Participants were told that choosing to go was the fastest option (paired with a positive chiming sound and blue tilde visual), but if a car passed through the intersection, they would crash and receive a 5‐s delay (paired with a honking car and crashing sounds, and broken windshield visual). Thus, go decisions reflect risky decision making given that the outcome is uncertain. A stop decision causes a 2.5‐s delay, which results in either a necessary stop (paired with an approaching honking car) or an unnecessary stop (no additional cues provided). As such, stop decisions represent safe decision making because the outcome is certain. Participants were trained on the YLG by playing two practice rounds before the scan, during which they received a warning (paired with an error sound and red X visual) and a 5‐s delay if they did not make a decision fast enough at an intersection to discourage nonresponses. Although unknown to the participants, the no‐decision trials were followed by a 1‐s delay during the scan rounds, which replaced the 5‐s delay present in practice rounds.

Younger siblings completed three rounds of the YLG during the fMRI scan, whereas older siblings completed one round, with each round lasting approximately 4 min. First, the older sibling completed the YLG (round A), which was recorded using the computer screen recording program (Bandicam, Seoul, Korea). Although during consent each family member was told that their responses may be shared with other family members, the older sibling was not explicitly made aware of the recording process as they completed the game. Next, the younger sibling completed two baseline rounds (rounds B and C) and then observed a video of their older sibling’s performance while in the scanner (i.e., social learning). Prior to beginning observation, the younger sibling was informed that their older sibling’s task performance had been recorded earlier in the session. They were explicitly instructed to not press any buttons, but to watch how their sibling played the game. Following this observation round, the younger sibling completed one final round (round D) of the YLG (see Figure 1b). Each round included 30 intersections in which the timing and onset of the yellow light was 1.5 s after the previous trial. The intersections varied in the perceived distance to the yellow light (200–250 feet) and had a 50% probability of a car passing through, although these details were not made explicit to the participants. Each practice and scan round of the YLG differed in its intersection conditions, including the onset of yellow and red lights as well as which intersections were dangerous versus safe.

### fMRI Data Acquisition

Brain images were collected using a research dedicated 3 Tesla Siemens Prisma MRI scanner. The YLG was presented on a computer screen and projected through a mirror. High‐resolution anatomical images, T1 magnetization‐prepared rapid‐acquisition gradient echo (MPRAGE; TR = 2,400 ms; TE = 2.22 ms; matrix = 256 × 256; FOV = 256 mm; 0.8 mm isotropic voxel; 208 slices), and T2‐weighted structural matched‐bandwidth (MBW; TR = 5,700 ms; TE = 65 ms; matrix = 192 × 192; FOV = 230 mm; 38 slices; slice thickness = 3 mm) were acquired first. T2*‐weighted echo‐planar imaging (EPI) volume (TR = 2,000 ms; TE = 25 ms; matrix = 92 × 92; FOV = 230 mm; 37 slices with 0.3 mm interslice gap; slice thickness = 3 mm; voxel size 2.5 × 2.5 × 3 mm^3^) was acquired during the YLG. The orientation for the EPI and MBW scans was oblique axial to maximize brain coverage and to reduce signal dropout.

### fMRI Data Preprocessing and Analysis

Preprocessing was carried out using FSL (FMRIB's Software Library, version 5.0.10; www.fmrib.ox.ac.uk/fsl). The following preprocessing was applied: motion correction using MCFLIRT (Jenkinson et al., 2002; all participants exhibited little head movement (>2.0 mm interslice movement on <10% of slices)); nonbrain removal using BET (Smith, 2002); and grand‐mean intensity normalization of the entire 4D dataset by a single multiplicative factor. Spatial smoothing was not applied as the focus of analysis was on the pattern of similarity of activation.

Participants’ brain response patterns during go decisions in the YLG were estimated at the individual level using the Statistical Parametric Mapping software package (SPM8; Welcome Department of Cognitive Neurology, Institute of Neurology, London, UK). To this end, the individual‐level GLMs were performed on adolescents’ native space using a fixed effects event‐related design with conditions of interest including go decisions, stop decisions, crashes, successful passes, necessary stops, unnecessary stops, and no decisions. The six motion parameters were included as nuisance regressors. Decision durations were modeled when the traffic light turned yellow (onset time) until the participant made a decision to go or stop. The duration of outcomes lasted 1 s following their onset cues (i.e., a blue tilde for successful passes, a broken windshield for crashes, and 2.5 s following the car stopping). The baseline (i.e., driving durations outside of the decision and outcome duration time frames) was not explicitly modeled and therefore serves as the implicit baseline for analyses. Individual‐level contrasts were utilized to compute linear contrasts images for the condition of interest, go decisions relative to baseline, to capture risky decision making. After the individual GLM, registration matrix and warping field were computed between functional images, the high‐resolution structural images (T1 and T2 anatomical images), and the standard Montreal Neurological Institute (MNI) 2‐mm brain using Advanced Neuroimaging Tools (ANTs; Avants et al., 2011). More specifically, older sibling EPI data were co‐registered to their corresponding anatomical images (T2 and T1; rigid body), and the T1 anatomical image was registered (affine) and warped to the standard Montreal Neurological Institute (MNI) 2‐mm brain. Younger sibling EPI data were also co‐registered to the anatomical images and warped to the MNI brain, but the older siblings’ MNI‐warped T1 image was utilized as a standard reference brain when the registration matrix and warping field were computed between anatomical and standard images. Using this registration matrix and warping field, estimated beta maps of individuals for go decisions were finally normalized onto the standard space. Our analyses were restricted to go decisions given our focus on examining the neural processes underlying older sibling influence on adolescent risky decision making. Five dyads were excluded from the neural analyses due to a participant in the dyad yielding less than 4 go decisions in a round (*n* = 38). This cutoff is a typical retention threshold that ensures sufficient data for reliable neural results to be estimated (Op de Macks et al., 2018).

**Figure 2:**
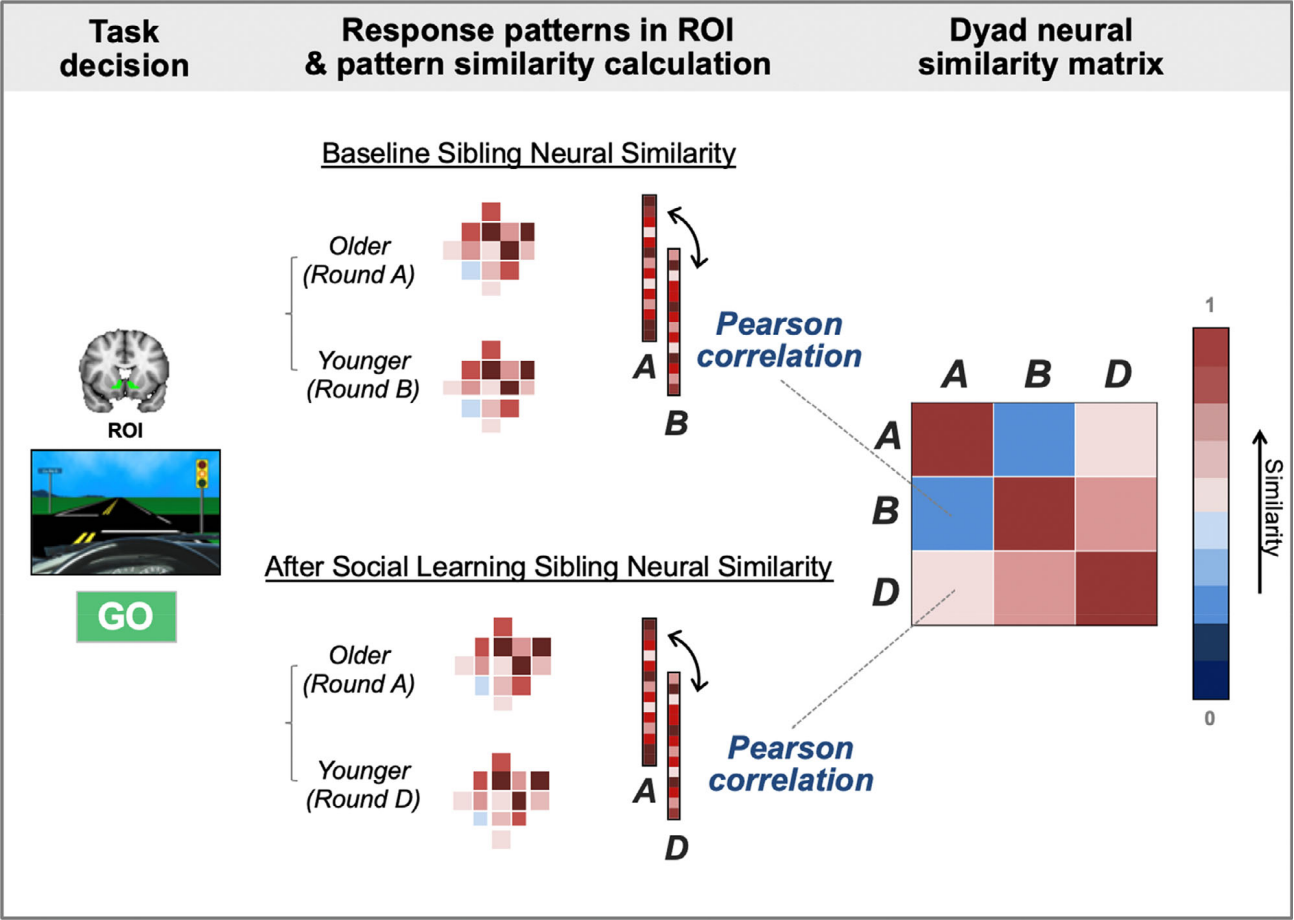
Representational similarity analysis procedure to test sibling neural pattern similarity at baseline and after social learning. These analyses were conducted for regions of interest (ROI; i.e., ventral striatum, anterior insula, and ventromedial prefrontal cortex) during go decisions of the Yellow Light Game. For each individual, ROI response patterns were obtained by extracting neural activation for each voxel and vectorizing these values. Once these vectors were computed for the older sibling (round A) and the younger sibling (round B and round D), pattern similarity was calculated by computing correlation coefficients between round A and round B for baseline sibling neural similarity and between round A and round D for sibling neural similarity after social learning.

For the neural similarity computations, we focused on the VS, AI, and vmPFC regions because these regions have been implicated in the process of adolescent risky decision making and reward valuation (e.g., Kahn et al., 2015; Peake et al., 2013), especially during social influence (e.g., Guassi Moreira & Telzer, 2018; Telzer, Ichien, et al., 2015; Telzer, Fuligni, et al., 2015;). To this end, anatomical masks were adopted to extract neural response patterns between siblings during go decisions. The bilateral VS (*k* = 342) and AI (*k* = 3,478) ROI masks were obtained from the WFUpickatlas (Maldjian et al., 2003; Tzourio‐Mazoyer et al., 2003). The vmPFC (*k = *2,011) was defined structurally using the frontal medial cortex Harvard‐Oxford atlas. For each ROI, we extracted neural activation for each voxel, vectorized them for computing pairwise *Pearson* correlation coefficients, and transformed coefficients to *z*‐value (*Fisher’s z transformation*) for subsequent statistical analyses (see Figure 2; Lee et al., 2017, 2018). Specifically, the pattern similarity between siblings in each dyad was computed for go decisions to establish baseline neural similarity between siblings, and postsocial learning neural similarity between siblings. These correlation coefficients were then used to test differences between baseline and postsocial learning neural similarity, as well as whether these similarities differed depending on individual differences in sibling modeling and differentiation. For display and interpretational purposes, figures report the similarity values as Pearson coefficients (*r*).

## Results

### Behavioral Results

Descriptive statistics of each round in the YLG are displayed in Table 2. Behavioral analyses focused on go decisions only, as stop decisions are the exact inverse of go decisions (with the exception of no decisions). We initially tested whether younger siblings’ risky decisions (go decisions) significantly changed across runs of the YLG, given that participants have been shown to exhibit learning effects on similar tasks (Kahn et al., 2015). We conducted a repeated measures analysis of variance including rounds B, C, and D, which showed significant differences between participants, *F*(1, 42) = 803.162, *p* < .001, but not across the runs, *F*(2, 58) = 2.089, *p* = .130. As such, subsequent analyses focus on round B as the younger siblings’ baseline in risky decision making given that round B was the first round of the YLG for the younger siblings in the scanner, just as round A was the first round of the YLG for older siblings, which made these rounds comparable as baselines between siblings. Nonetheless, we ran parallel analyses with round C to show that differences found following social learning were not due to time or more practice.

**Table 2:** Descriptive Statistics for Go Decisions Across Rounds of the YLG

Go Decisions	A	B	C	D
Range	0−19	0−23	4−28	4−30
*M*	10.98	12.49	13.95	14.63
*SD*	4.17	5.11	5.02	5.30

Note.

*M* = mean; *SD* = standard deviation.

Note that stop decisions are the exact inverse of go decisions (with the exception of no decisions) so are not included in the descriptive statistics.

**Figure 3:**
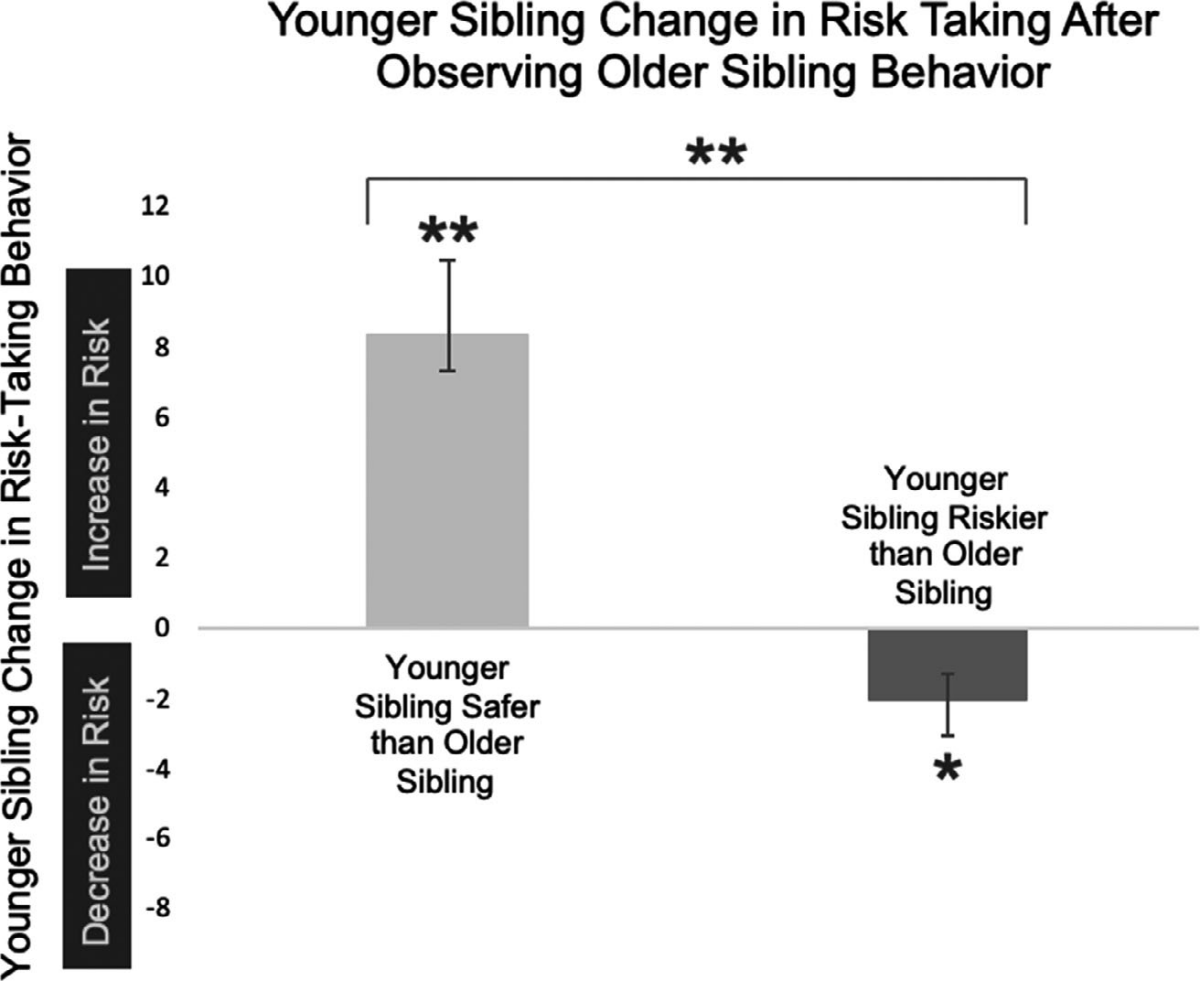
Change in risk‐taking behavior (go decisions) was significantly different between adolescents who were safer than their older siblings and adolescents who were riskier than their older siblings. Younger siblings who were safer than older siblings became riskier, whereas younger siblings who were riskier than older siblings became safer. *p < .05, **p < .01.

To examine whether adolescent risk‐taking behavior changes after social learning, younger siblings were split into two groups based on their behavior during round B: (1) younger siblings who took less risks than older siblings at baseline (*n* = 17) and (2) younger siblings who took more risks than older siblings at baseline (*n* = 23). Three sibling dyads were excluded from this behavioral analysis because the younger sibling and older sibling took the same amount of risks at baseline. We computed a difference score for the younger siblings’ risk behavior, which represents their change in go decisions between round B and round D, such that higher scores represent increases in risk behavior, whereas negative scores represent decreases in risk behavior. We ran an independent samples test (equal variances assumed) comparing the two groups, which showed that younger siblings who were safer than their older siblings and younger siblings who were riskier than their older siblings were significantly different in the degree to which their risk behavior changed after social learning from older siblings, *t*(38) = 5.144, *p* < .001, 95% CI [6.332, 14.548], Cohen’s *d* = 1.485; Figure 3). In particular, one‐sample *t* tests relative to 0 (i.e., representing no change in behavior) indicated that younger siblings who were initially safer than older siblings became significantly risker, *t*(16) = 3.98, *p* < .01, 95% CI [3.908, 12.798], *d* = 0.966), whereas younger siblings who were initially riskier than their older siblings became significantly safer, *t*(22) = −2.57, *p* < .05, 95% CI [−3.771, −0.403], *d* = −0.536) in their decision making after observing the older sibling performance.

**Figure 4:**
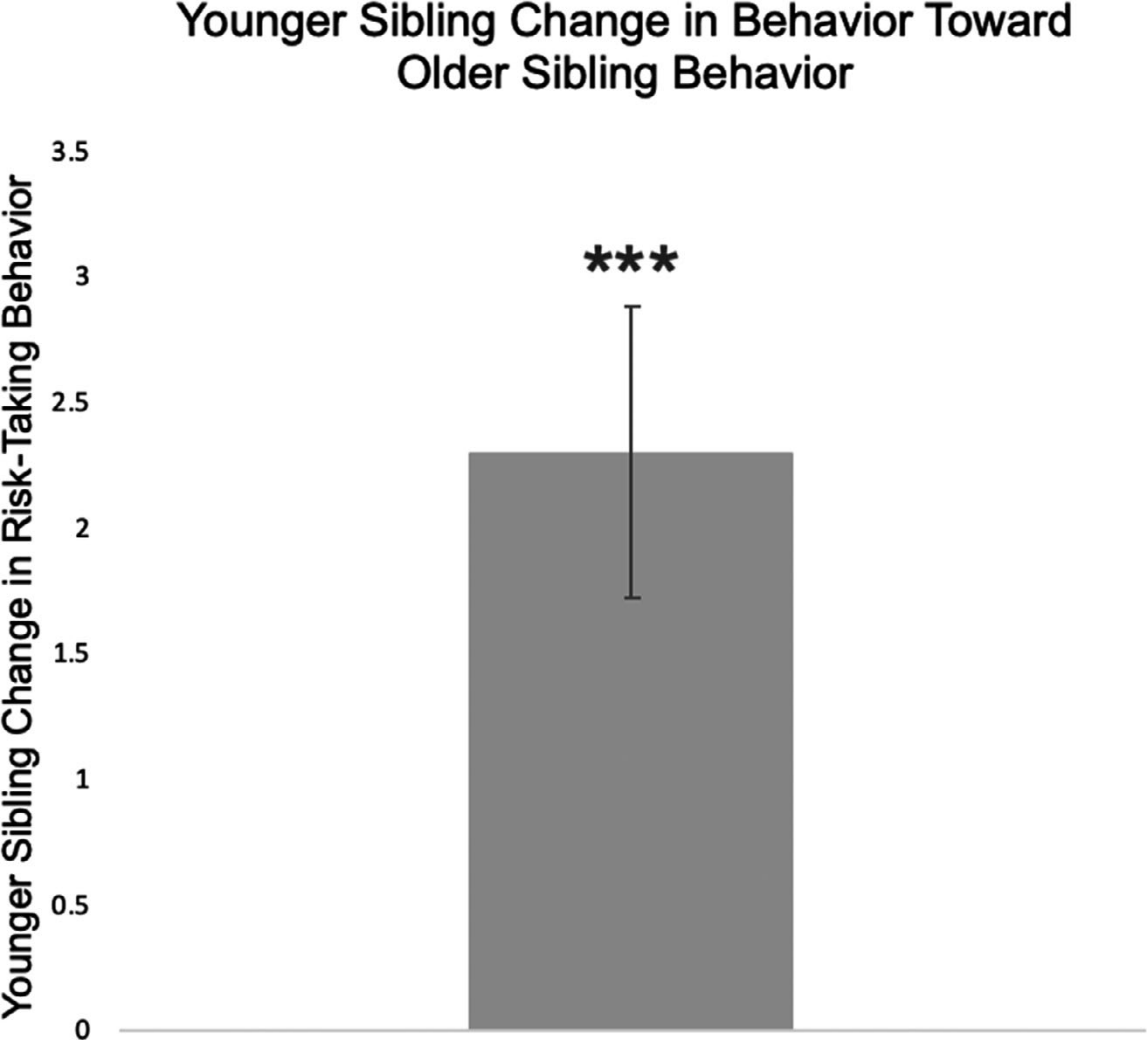
Younger sibling risk‐taking behavior (go decisions) changed significantly in the direction of older sibling risk‐taking behavior. ***p < .001.

Next, we calculated the overall social influence effect by calculating the absolute value of change in the younger siblings’ risk behavior from round B to round D. If a younger sibling shifted their behavior away from that of their older sibling, the resulting social influence score was negative, whereas if a participant shifted their behavior toward that of their older sibling, the resulting social influence score was positive. Positive influence was capped at a maximum determined by the difference between the older sibling’s behavior and the younger sibling’s change in behavior (i.e., an influence score could not be greater than the total initial difference between younger sibling and older sibling). This metric is consistent with prior work on social influence (e.g., Welborn et al., 2016). A one‐sample *t* test including the full sample showed that younger sibling risk‐taking behavior significantly changed in the direction of their older sibling after observing their older siblings take risks, *t*(42) = 3.95, *p* < .001, 95% CI [1.127, 3.478], *d* = 0.603. On average, younger sibling behavior changed by 2.3 risky decisions (*SD* = 3.8; Figure 4). This finding suggests that regardless of baseline risk‐taking behavior, younger siblings become more similar to their older siblings in risk‐taking behavior after observing their performance.

We also assessed whether change in adolescent risky decisions toward older sibling behavior would be greater for younger siblings who reported higher older sibling modeling and lower differentiation from their older sibling. Younger sibling change in risky decisions toward older sibling behavior was regressed onto modeling and differentiation, respectively. Both modeling and differentiation were nonsignificant predictors of younger sibling change in risky behavior toward older sibling behavior (*B* = −1.36, *p =* .13; *B* = 0.55, *p =* .54).

### fMRI Results

Sibling similarity during risky decisions at baseline and after social learning was examined at the level of the brain by examining similarity between younger and older siblings in patterns of brain activation within each ROI. To calculate similarity in patterns of activation, we extracted the neural activation for each voxel and ordered the values as vectors to compute pairwise *Pearson* correlation coefficients (see Figure 2). Coefficients were obtained for each dyad’s neural pattern similarity in the VS, vmPFC, and left and right AI, at baseline (*r*_AB_) and postsocial learning (*r*_AD_). This coefficient represents the correlation between younger and older sibling dyads in their neural patterns within each ROI during risky decision making. To examine change in neural pattern similarity following social learning, we conducted paired samples *t* tests between the coefficients for baseline and postsocial learning for each ROI. Our primary analyses first tested these associations during go decisions.

Neural pattern similarity between younger and older siblings during go decisions did not significantly increase on average from baseline (*r*_AB_) to postsocial learning (*r*_AD_; VS: *t =* −0.154, *p =* .879, 95% CI [−0.050, 0.042], *d =* −0.032; vmPFC: *t =* −0.869, *p =* .391, 95% CI [−0.060, 0.024], *d =* −0.191; left AI: *t =* −0.335, *p =* .740, 95% CI [−0.034, 0.025], *d =* −0.081; right AI: *t =* −0.128, *p =* .899, 95% CI [−0.035, 0.030], *d =* −0.033). Neural pattern similarity between younger and older siblings also did not significantly increase on average across the two baseline rounds (*r*_AB_, *r*_AC_; VS: *t =* 1.166, *p =* .251, 95% CI [−0.020, 0.075], *d =* 0.235; vmPFC: *t =* −0.148, *p =* .883, 95% CI [−0.071, 0.064], *d =* −0.033; left AI: *t =* −0.887, *p =* .381, 95% CI [−0.048, 0.019], *d =* −0.193; right AI: *t =* 0.510, *p =* .613, 95% CI [−0.028, 0.047], *d =* 0.133).

**Figure 5:**
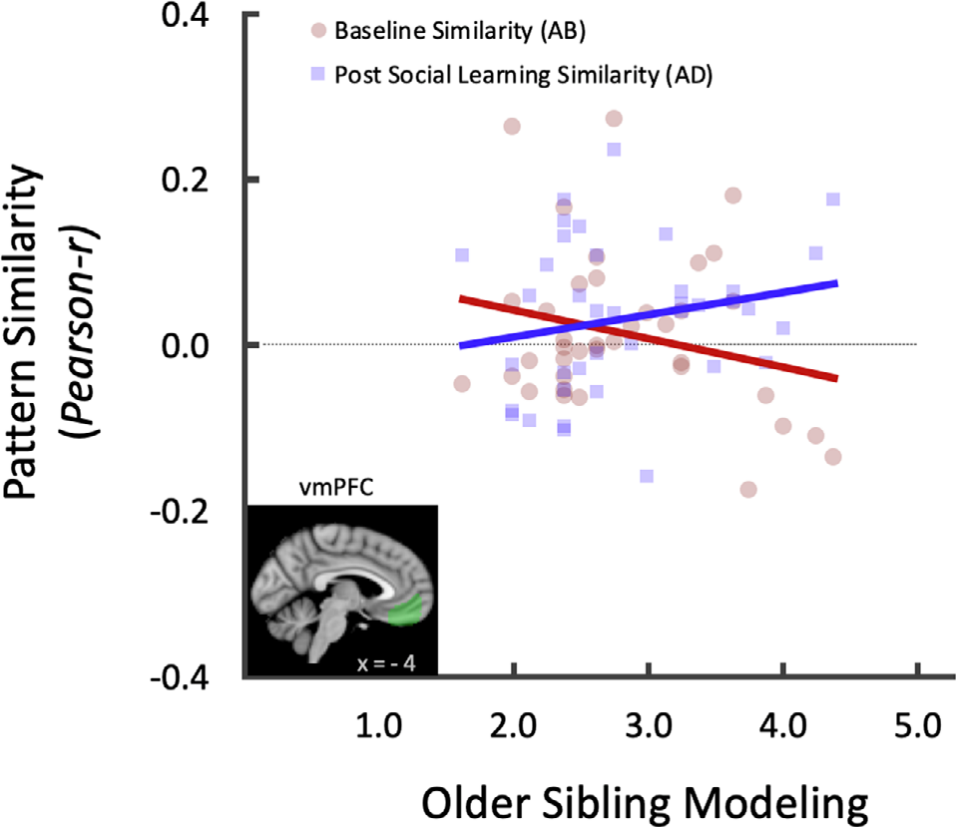
Slopes represent the correlation between sibling neural similarity during go decisions and adolescent report of older sibling modeling. The difference between the two slopes is significantly different, indicating that neural similarity during risky decision making between siblings is significantly different between baseline and postsocial learning similarity, in relation to perceptions of older sibling modeling.

Next, we examined individual differences in modeling and differentiation to test whether younger siblings reporting greater older modeling and less differentiation from their older siblings showed greater increases in neural pattern similarity following social learning. To this end, we correlated modeling and differentiation with the neural pattern similarity coefficients obtained at baseline (*r*_AB_) and after social learning (*r*_AD_) for each ROI. These correlation coefficients were converted into *z*‐scores using Fisher’s *r*‐to‐*z* transformation for the estimation of the asymptotic covariance matrix, and then, z tests were conducted to test the mean difference between the correlations (Lee & Preacher, 2013). We performed one‐tailed tests, because we hypothesized a priori that adolescents reporting higher modeling and less differentiation would show greater increases in neural pattern similarity following social learning. The correlation between modeling and sibling neural similarity in the vmPFC significantly differed between baseline and postsocial learning (*z =* −2.01, *p =* .022, Cohen’s *q =* .455; Figure 5). Although the correlations between modeling and baseline neural similarity (*r =* −.24, *p =* .147) or postsocial learning neural similarity (*r =* .207, *p =* .212) were not significant, the difference between these correlations was significant. These results suggest that for adolescents who perceive their older sibling as an excellent model, observing their older sibling take risks is associated with greater increases in similarity in neural patterns in the vmPFC between siblings. Sibling modeling did not significantly modulate dyadic neural similarity in the VS (*z =* −0.356, *p =* .36, *q =* .194) or AI (left: *z =* 1.414, *p =* .079, *q =* .356; right: *z =* 0.61, *p =* .271, *q =* .161) between baseline and postsocial learning.

**Figure 6:**
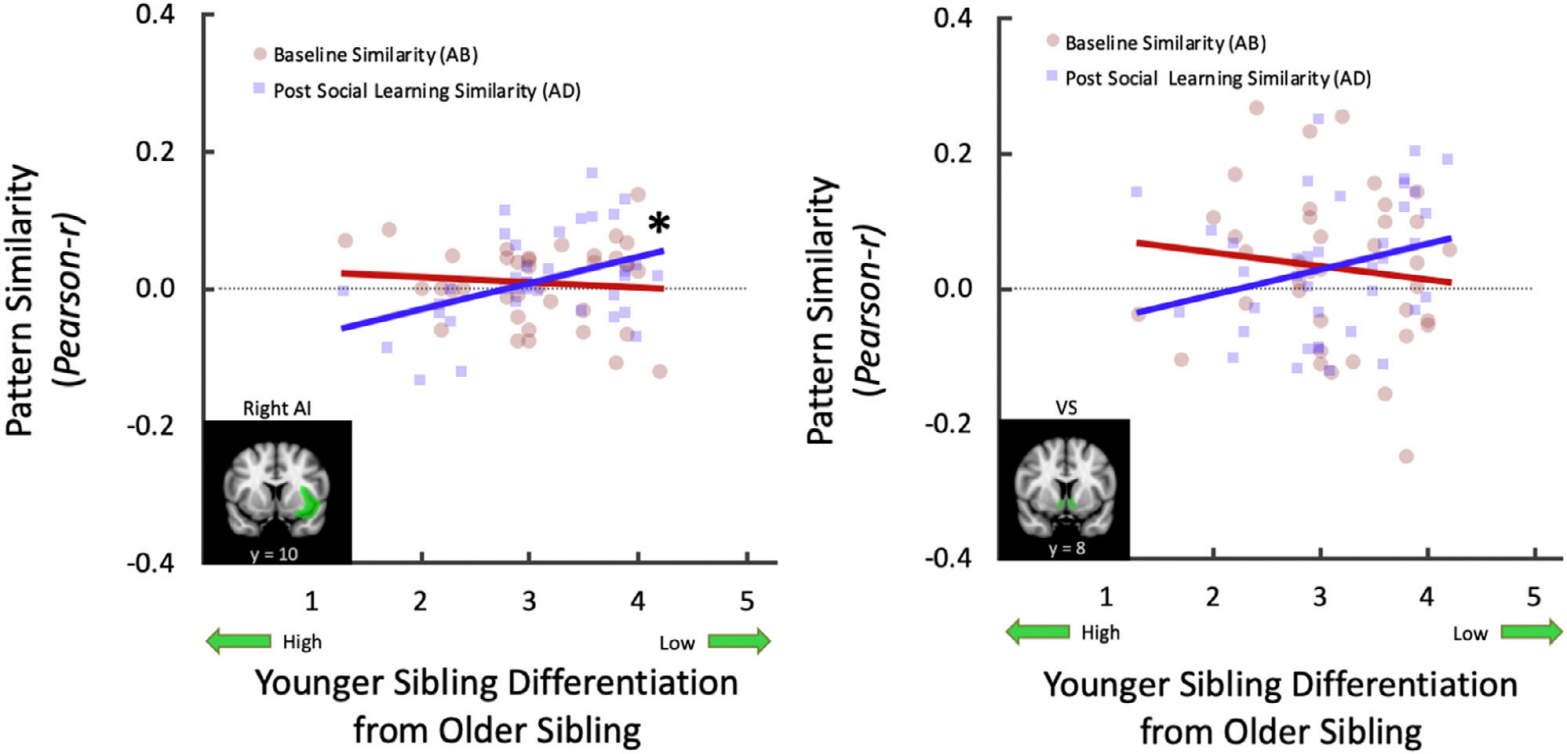
Slopes represent the correlation between sibling neural similarity and adolescent report of differentiating from their older sibling. The difference between the two slopes was significantly different, indicating that neural similarity during risky decision making between siblings is significantly different after adolescents observe older sibling performance, compared to baseline neural similarity between siblings, in relation to perceptions of differentiation. *p < .05.

The correlation between differentiation and sibling neural similarity in both the AI and VS significantly differed between baseline and postsocial learning (AI: *z =* −2.03, *p =* .021, *q =* .538; VS: *z =* 1.69, *p =* .046, *q =* .386; Figure 6). Adolescents reporting lower differentiation showed significantly greater neural similarity in the right AI postsocial learning (*r =* .42, *p =* .010), but not at baseline (*r =* −.10, *p =* .576), indicating that the less adolescents actively differentiate from their older siblings, the more they recruit similar patterns of activity in the AI to their older sibling after social learning. Neural similarity in the VS did not significantly correlate with differentiation at baseline (*r =* −.12, *p =* .474) or postsocial learning (*r =* .26, *p =* .116), despite the significant difference between the two correlations. Differentiation was not significantly associated with differences in dyadic neural similarity in the vmPFC (*z =* 0.067, *p =* .473, *q =* .001) between baseline and postsocial learning.

To examine whether these findings were unique to the social learning process, we tested whether sibling modeling and differentiation were associated with changes in neural similarity across the baseline runs (*r*_AB_, *r*_AC_). Sibling modeling and differentiation were not correlated with significant differences in sibling neural similarity across the baseline rounds (left AI: *z =* 0.221, *p =* .412, *q =* .051; *z =* −0.324, *p =* .373, *q =* .073; right AI: *z =* 1.289, *p =* .099, *q =* .343; *z =* −0.237, *p =* .407, *q =* .063; vmPFC: *z =* 0.436, *p =* .331, *q =* .209; *z =* −0.508, *p =* .306, *q =* .085; VS: *z =* 1.052, *p =* .146, *q =* .221; *z =* −1.272, *p =* .102, *q =* .266, respectively). Together, these findings indicate that younger siblings do not become more neurally similar to their older siblings in AI, vmPFC, or VS during go decisions if they do not observe their older siblings’ behavior, particularly as modulated by modeling and differentiation.

Finally, we examined whether these processes were specific to risky decision making by conducting the same analyses using stop decisions. We conducted paired sample *t* tests to assess sibling neural similarity during safe decision making at baseline and after social learning for each ROI. Neural pattern similarity between younger and older siblings during stop decisions did not significantly increase on average from baseline (*r*_AB_) to postsocial learning (*r*_AD_; VS: *t =* −1.473, *p =* .149 95% CI [−0.089, 0.014], *d =* −0.344; vmPFC: *t =* 1.022, *p =* .313, 95% CI [−0.022, .067], *d =* 0.206; left AI: *t =* −1.194, *p =* .240, 95% CI [−0.050, 0.013], *d =* −0.254; right AI: *t =* 0.293, *p =* .771, 95% CI [−0.031, 0.041], *d =* 0.067). In addition, we tested whether sibling modeling and differentiation were associated with changes in neural similarity between baseline and postsocial learning. The correlations between sibling neural similarity and sibling individual differences were insignificant for modeling (VS: *z =* 0.401, *p =* .344, *q =* .096; vmPFC: *z =* −0.024, *p =* .490, *q =* .005; left AI: *z =* −0.412, *p =* .340, *q =* .092; right AI: *z =* 0.642, *p =* .261, *q =* .030) and differentiation (VS: *z =* −1.030, *p =* .152, *q =* .247; vmPFC: *z =* −0.639, *p =* .262, *q =* .137; left AI: *z =* 1.491, *p =* .068, *q =* .331; right AI: *z =* 0.326, *p =* .372, *q =* .078).

## Discussion

Siblings are a significant social influence on adolescent risk taking (Slomkowski et al., 2005; Whiteman et al., 2013, 2014) and, as such, represent an ideal relationship to investigate social influence on risk taking. Given the strides social neuroscience has taken to inform our understanding of adolescent risky decision making (Somerville et al., 2010; Steinberg, 2008; Telzer et al., 2017), we conducted an exploratory experimental study to examine the effects of observation, a salient stage in social learning, on both brain and behavior. Our preliminary findings show that older siblings’ behavior has the potential to influence adolescents’ decisions to engage in, or abstain from, risk taking following observation, and this is represented in the brain via shared patterns of neural processing of risky decision making in the VS, AI, and vmPFC. Furthermore, sibling neural similarity following observation differed depending on the sibling influence mechanisms of modeling and differentiation. Together, our preliminary findings identify the VS, AI, and vmPFC as brain regions implicated in social influence susceptibility, as well as highlight siblings as salient social agents in how adolescents process risky decision making.

Our findings show that adolescents become more similar to their older sibling in risk‐taking behavior after social learning, corroborating previous behavioral studies using longitudinal and multiple‐informant methods (Craine et al., 2009; Defoe et al., 2013; Duncan et al., 2001; Duncan, Duncan, & Hyman, 1996; Slomkowski, Rende, Conger, Simons, & Conger, 2001; Stormshak et al., 2004; Whiteman et al., 2013; Whiteman, Zeiders, Killoren, Rodriguez, & Updegraff, 2014). In particular, younger siblings who were riskier compared to their older sibling became safer, whereas younger siblings who were safer compared to their older sibling became riskier. Thus, older siblings have the potential to influence the risky behavior of their younger siblings, for better and for worse. However, adolescent behavior did not change as a function of modeling or differentiation, possibly due to the effect of observation on behavior. Specifically, most adolescents changed their behavior after watching their older siblings for only a few minutes, highlighting the association between observation and adolescent behavior, above and beyond relationship processes. This manipulation provides preliminary evidence of behavioral changes due to social learning from a salient and familiar social agent. Although research has examined explicit forms of social influence on adolescent decision making, such as physical presence (e.g., Chein et al., 2011; Guassi Moreira & Telzer, 2018), expressed attitudes or decisions (Cascio et al., 2015; van Hoorn, van Dijk, Guroglu, & Crone, 2016; Welborn et al., 2016; Whiteman et al., 2016), and relationship quality (e.g, Qu et al., 2015; Telzer, Ichien, et al., 2015; Telzer, Fuligni, et al., 2015), risk taking is heavily influenced by observing behavior and internalizing the norms set by others to implement in future behaviors (e.g., Pomery et al., 2005; Snyder et al., 2005; Whiteman et al., 2014). Sex composition, shared environment (e.g., common interests, parenting), and relationship quality among sibling dyads oftentimes amplify the effects of modeling and differentiation (e.g., Whiteman et al., 2010, 2007a; Whiteman, McHale, & Crouter, 2007b) and thus represent an opportunity for future work to better understand modeling and differentiation as unique social learning processes. These findings highlight the potential for older siblings to provide both risky and cautious models of behavior during adolescence and provide preliminary support for social learning theory as a way through which adolescents process social influences.

Interestingly, we did not find that adolescents’ neural patterns became more similar to their siblings on average after social learning. Instead, adolescent neural patterns only became more similar to their older sibling to the extent that they perceived high modeling and low differentiation, underscoring that the way in which individuals process information following social learning does not occur identically for everyone. In fact, adolescent behavior was influenced by the risky behavior of their older sibling, whereas the neural processes recruited after social learning varied based on characteristics of the sibling dyad. Indeed, we found that higher older sibling modeling and lower adolescent differentiation were associated with greater neural similarity between siblings following social learning, compared to baseline, corroborating previous work (Whiteman et al., 2013), and consistent with theory suggesting that individuals internalize the norms of others when they are close and more similar to each other (Akers et al., 1979; Bandura, 1977). Thus, adolescents appear to generally adjust their behavior to become more similar to their older siblings following observation, whereas changes in sibling neural similarity may be more discerning, and in part reliant on adolescent perceptions of sibling dyad characteristics.

At the neural level, we found that the vmPFC may be implicated in the sibling influence mechanism of modeling, such that perceiving an older sibling as a good model was associated with adolescents showing more neural similarity in the vmPFC to their older sibling during risky decision making after observing them take risks. This finding suggests that older siblings who model for their younger siblings can be a salient influence on the way in which adolescents neurobiologically process risky decision making, specifically in greater shared recruitment of a brain region associated with self‐determined choice (Murayama et al., 2015) and valuation of rewards (for a review, Blakemore & Robbins, 2012; Hare, Camerer, & Rangel, 2009). Thus, autonomous decision making and valuing older sibling’ behaviors may be pertinent to modeling as an underlying mechanism of social learning. Furthermore, given that modeling is a behavioral social learning mechanism that transmits expectations and engagement in health risk behaviors from older to younger siblings (e.g., Whiteman et al., 2014), the vmPFC may be pivotal in emulating risky decision making from salient models during adolescence.

The VS and AI were recruited in relation to the sibling influence mechanism of differentiation. Adolescents who did not try to differentiate from their older sibling in their everyday lives showed more neural similarity in the VS and AI during risky decision making to their older sibling after social learning. Given that the VS is implicated in reward processing and risky decision making during adolescence (Kahn et al., 2015; Van Leijenhorst et al., 2010), the VS likely serves an analogous function for differentiation. Heightened striatal activity exhibited during adolescence predisposes individuals to explore and take risks during salient experiences (Telzer, 2016), and in this case, adolescents who do not feel the need to differentiate may experience enjoyment in risky decision making more similarly to how their older sibling experiences it. The AI is also associated with risky decision making during adolescence, specifically in detecting the salience of making risky decisions (Smith et al., 2014; Telzer, Ichien, et al., 2015; Telzer, Fuligni, et al., 2015). As such, integrating relevant information to make decisions, such as observing older sibling behavior, may also underlie the process of differentiation following social learning and thus relate to greater sibling neural similarity when differentiation is low. Differentiation is a psychological process in which individuals consciously and unconsciously select different activities that contrast themselves from a sibling (Whiteman et al., 2013), particularly toward identity development during adolescence (McHale et al., 2012). Because the findings link low differentiation to greater sibling neural similarity after observation in the VS and AI, these regions may tap into detecting the rewarding and salient nature of potentially replicating or distinguishing from a model’s behavior. Together, these preliminary findings identify brain regions associated with social influence processes following social learning and highlight siblings as an important influence during adolescence.

This exploratory study is the first to examine the effects of older sibling risk taking on adolescent risk taking, behaviorally and neurobiologically, and given its exploratory nature, replication is warranted to test whether any of the findings were false positives. In addition, it brings to light promising directions toward our understanding of the neural underpinnings of social influence susceptibility and the influence of siblings on adolescent risky decision making. First, this study used the real behavior of older siblings to examine sibling influence on adolescent risk taking. Even though this approach provides ecological validity in examining how a salient and familiar social agent may influence adolescent decision making, it limited the variance of older sibling risk taking and the ability to test controlled differences between risky and cautious social models. Future work would benefit from using fixed conditions of older sibling performance, such as high risk‐takers and low risk‐takers, to investigate whether adolescents become riskier or more cautious depending on the condition observed. Alternatively, predictive models using machine learning would be advantageous in identifying whether similarity at the level of behavior and the brain results in similarity in psychological processes (Varoquaux & Poldrack, 2018), including reward processing and saliency detection during risky decision making. Second, previous work has shown that sibling relationship quality and sex composition of dyads modulate whether adolescents emulate the risky behavior of their older sibling (e.g., Bank, Burraston, & Snyder, 2004; Rowe et al., 1992; Slomkowski et al., 2001), such that warmth between sisters and hostility between brothers exacerbates modeling and similarity in risk‐taking behavior. Age spacing may serve as an additional moderator, given that concordance in risk behaviors is often higher for siblings who are closer in age (Trim, Leuthe, & Chassin, 2006). Future work would benefit from recruiting a larger sample to disentangle whether these factors influence similarity in neural activation patterns during risky decision making. Relatedly, the current study investigated the unidirectional influence of older siblings on younger sibling. Future work should utilize a bidirectional framework to examine if older siblings may similarly model their younger counterparts. Third, the majority of the sibling dyads were full biological siblings, and as such, future work should utilize genetically informed designs to test the relative contribution of shared genetics and environment to sibling behavioral and neural similarity. Last, we propose that these findings generalize to other social influence partners, and we utilized siblings as an ideal model for examining the social influence process. Although we found similarity in neural patterns between adolescent siblings, an important avenue of future work should examine social learning via manipulations with peers and other salient social models (known and unknown), different types of behavior (e.g., prosocial), as well as control conditions given that social desirability may have played a role in adolescent decisions to conform to their older sibling’s behavior. Of note, one‐tailed tests were used given our *a priori* hypotheses that younger siblings would become more similar to their older siblings at the level of behavior and the brain after social learning, and as such, the findings should be interpreted with caution, particularly given the limited power due to the small sample size.

In conclusion, the current study provides novel preliminary evidence on the importance of social learning on adolescent risk taking. Using a social learning design, we demonstrated that adolescents can learn about risk taking from their siblings. In addition, we identified neural processes of social influence susceptibility in relation to risky decision making. Findings from this exploratory study contribute toward our understanding of social learning, and the significant role it plays in adolescent risk taking. Given the power of social influence on risky decision making during adolescence, we encourage adolescent researchers to consider where important social figures *lead*, such as older siblings, and what mechanisms contribute to whether adolescents will *follow*.
